# Tracheostomy in childhood: review of the literature on complications and mortality over the last three decades^☆^

**DOI:** 10.1016/j.bjorl.2016.04.005

**Published:** 2016-05-06

**Authors:** Ana Paula Ligoski Dal’Astra, Ariane Vieira Quirino, Juliana Alves de Sousa Caixêta, Melissa Ameloti Gomes Avelino

**Affiliations:** a Pontifícia Universidade Católica de Goiás, Goiânia, GO, Brazil; b Centro Universitário de Anápolis (UniEvangélica), Anápolis, GO, Brazil; c Universidade Federal de Goiás, Goiânia, GO, Brazil

**Keywords:** Tracheostomy, Child, Mortality, Complication, Traqueostomia, Criança, Mortalidade, Complicação

## Abstract

**Introduction:**

Tracheostomy is a procedure with unique characteristics when used on pediatric patients due to the greater technical difficulty and higher morbidity and mortality rates relative to the procedure in adults. In recent decades, there have been significant changes in the medical care available to children, particularly for those who need intensive care. Surgical conditions have also improved, and there has been an advent of new equipment and medications. These advances have brought changes to both tracheostomy indications and tracheostomy complications.

**Objective:**

To perform a review of the articles published over the last three decades on the complications and mortality associated with tracheostomies in children.

**Methods:**

Articles were selected from the Cochrane, Latin American and Caribbean Health Sciences Literature, SciELO, National Library of Medicine (Medline Plus), and PubMed online databases. The articles selected had been published between January 1985 and December 2014, and the data was compared using the Chi-square test.

**Results:**

A total of 3797 articles were chosen, 47 of which were used as the basis for this review. When the three decades were evaluated as a whole, an increase in tracheostomies in male children under one year of age was found. The most common complications during the period analyzed in descending order of frequency were granuloma, infection, and obstruction of the cannula, accidental decannulation, and post-decannulation tracheocutaneous fistula. In the second and third decades of the review, granulomas represented the most common complication; in the first decade of the review, pneumothoraces were the most common. Mortality associated with tracheostomy ranged from 0% to 5.9%, while overall mortality ranged from 2.2% to 59%. In addition, the review included four studies on premature and/or very underweight infants who had undergone tracheostomies; the studies reported evidence of higher mortality in this age group to be largely associated with underlying diseases.

**Conclusion:**

Improved surgical techniques and intensive care, the creation of new medications, and vaccines have all redefined the main complications and the mortality rates of tracheostomy in children. It is a safe procedure that increases chances of survival in those who require the prolonged use of mechanical ventilation.

## Resumo

**Introdução:**

A traqueostomia é um procedimento com características exclusivas quando usada em pacientes pediátricos devido à maior dificuldade técnica e maiores taxas de morbidade e mortalidade em relação ao procedimento em adultos. Nas últimas décadas, houve mudanças significativas na assistência médica disponível para crianças, especialmente para aqueles que necessitam de cuidados intensivos. As condições cirúrgicas também melhoraram e houve um advento de novos equipamentos e medicamentos. Estes avanços trouxeram mudanças tanto para as indicações de traqueostomia como para as suas complicações.

**Objetivo:**

Realizar uma revisão dos artigos publicados ao longo das últimas três décadas sobre as complicações e mortalidade em crianças associadas a traqueostomia.

**Método:**

Os artigos foram selecionados das bases de dados online da Cochrane, Latin American and Caribbean Health Sciences Literature, SciELO, National Library of Medicine (Medline Plus) e PubMed. Os artigos selecionados foram publicados entre janeiro de 1985 e dezembro de 2014 e os dados foram comparados com uso de teste do Chi-quadrado.

**Resultados:**

Foram selecionados 3.797 artigos, 47 dos quais foram usados como base para esta revisão. Quando as três décadas foram avaliadas como um todo, encontrou-se um aumento das traqueostomias em crianças do sexo masculino com menos de um ano de idade. As complicações mais comuns no período analisado, em ordem decrescente de frequência, foram granuloma, infecção, obstrução da cânula, decanulação acidental e fístula traqueocutânea pós-decanulação. Na segunda e terceira décadas de revisão, os granulomas representaram a complicação mais comum; na primeira década da revisão, pneumotórax foram as mais comuns. A mortalidade associada à traqueostomia variou de 0% a 5,9%, enquanto que a mortalidade global variou de 2,2% a 59%. Além disso, a revisão incluiu quatro estudos sobre lactentes prematuros e/ou nascidos com muito baixo peso que tinham sofrido traqueostomias; os estudos relataram evidências de que a maior mortalidade nessa faixa etária era em grande parte associada a doenças subjacentes.

**Conclusão:**

A melhoria das técnicas cirúrgicas e de cuidados intensivos, a criação de novos medicamentos e vacinas redefiniram as principais complicações e as taxas de mortalidade da traqueostomia em crianças. É um procedimento seguro que aumenta as chances de sobrevida naqueles que requerem o uso prolongado de ventilação mecânica.

## Introduction

Tracheostomies consist of a surgical opening of the trachea and the insertion of a tube that allows for the trachea to have a direct exchange with the external environment.^1^, ^2^, ^3^, ^4^ It is one of the oldest known surgical procedures; it was first described in 100 AD by Asclepiades in Ancient Greece. It came to be used more frequently in routine medicine in the mid-nineteenth century when Armand Trousseau employed the technique to treat many patients with dyspnea associated with diphtheria.^1^, ^2^, ^4^, ^5^, ^6^, ^7^, ^8^, ^9^

In recent decades, the tracheostomy has changed in terms of its indications, its complications, and the epidemiological profile of the patients that undergo this procedure. These changes are attributed to the development of new intensive care techniques, as well as to alterations in the epidemiology of infectious diseases, the increase in premature newborn survival rates, and the increase in the survival rates of newborns with birth defects.^2^, ^10^, ^11^, ^12^, ^13^, ^14^, ^15^, ^16^

This procedure is technically more difficult in pediatric patients relative to their adult counterparts, since children's trachea are smaller and softer and because the surgical space is more limited. In addition, some risks to the procedure are directly linked to age, and more specific such as premature birth, low birth weight, the duration of the tracheostomy, and associated serious underlying diseases. Thus, this group experiences higher morbidity and mortality rates than adult patients.^1^, ^2^, ^4^, ^5^, ^7^, ^10^, ^15^, ^17^, ^18^

The objective of this study was to perform a review of the literature from the last three decades in order to trace a profile of complications and mortality rates of tracheostomies in children, and also to determine whether this profile has changed between decades.

## Methods

A review of the literature was performed using the Cochrane, Latin American and Caribbean Health Sciences Literature (LILACS), SciELO, National Library of Medicine (Medline Plus), and PubMed databases. It included articles published between January 1985 and December 2014. After the articles were read, a review of the articles’ bibliographical references was performed in order to identify other potentially relevant studies.

In the Medline and PubMed databases, the MeSH terms “tracheotomy” and “tracheostomy” were used; they were associated with “complications”, “child”, and “mortality” as qualifiers. Meanwhile, in the SciELO, LILACS, and Cochrane databases, associations between the terms “tracheostomy/tracheotomy”, “complication”, “mortality” and “pediatric/child” were used. The search was limited to articles written in Portuguese, Spanish, and English and to a patient age group of 0–18 years of age.

The articles were independently evaluated by two of our study's authors. Articles that represented case reports and case series were excluded. Articles that did not contain sufficient data to evaluate complications and mortality in children that had undergone tracheostomies were also excluded.

The data was organized in Excel^®^ and analyzed using the Statistical Package for Social Sciences (SPSS^®^) software, version 21.0. The data from each decade was compared to the data from the other decades using the Chi-square test. The Confidence Interval was set at 95%, and values were considered significant when *p* < 0.05.

## Results

A total of 3797 articles were identified using the MeSH terms and the key words outlined in the methodology. From these articles, studies that included an age group of 0–18 years of age, were written in Portuguese, English, or Spanish, and were published between January 1985 and December 2014 were included; there were a total of 595 articles. One hundred and 32 articles that represented case reports and case series were excluded. After another review for relevance by two independent otolaryngologists, 547 articles were excluded and 47 articles remained.^1^, ^2^, ^3^, ^4^, ^5^, ^6^, ^7^, ^8^, ^9^, ^10^, ^11^, ^12^, ^13^, ^14^, ^15^, ^16^, ^17^, ^18^, ^19^, ^20^, ^21^, ^22^, ^23^, ^24^, ^25^, ^26^, ^27^, ^28^, ^29^, ^30^, ^31^, ^32^, ^33^, ^34^, ^35^, ^36^, ^37^, ^38^, ^39^, ^40^, ^41^, ^42^, ^43^, ^44^, ^45^, ^46^, ^47^ Most of the papers excluded at this time did not have sufficient data for review. Of these 47 articles, five studies were published between 1985 and 1994, 15 were published between 1995 and 2004, and 27 were published between 2005 and 2014. The distribution of the number of articles and the total number of patients per decade is shown in Table 1.

**Table 1:** Distribution of pediatric patients and tracheostomy studies by decade 1985–1994, 1995–2004, and 2005–2014.

Decade	Number of studies	Number of patients
1985–1994	5	480
1995–2004	15	1150
2005–2014	27	4303

Total	47	5933

### Epidemiology

Four studies considered only premature and/or severely underweight newborns.^19^, ^20^, ^21^, ^22^ Considering gender, tracheostomy is mainly performed in males.^1^, ^2^, ^3^, ^4^, ^5^, ^6^, ^7^, ^8^, ^9^, ^10^, ^11^, ^12^, ^13^, ^14^, ^15^, ^16^, ^18^, ^19^, ^20^, ^21^, ^22^, ^23^, ^24^, ^25^, ^26^, ^27^, ^28^, ^29^, ^30^, ^31^, ^32^, ^33^, ^34^, ^35^, ^36^, ^37^, ^38^, ^40^, ^42^, ^43^, ^45^ In the other articles, the number of children younger than one year of age relative to the other age groups ranged from 4% to 78%, as detailed in Table 2.^1^, ^2^, ^3^, ^6^, ^7^, ^8^, ^9^, ^12^, ^13^, ^14^, ^15^, ^16^, ^17^, ^18^, ^23^, ^26^, ^27^, ^28^, ^29^, ^34^, ^37^, ^38^, ^39^, ^40^, ^41^, ^42^, ^43^

**Table 2:** Data on the epidemiology and mortality of children who received a tracheostomy between 1985 and 2014.

Author	Year of publication	*n*	<1 year-old *n* (%)	Male *n* (%)	Mortality by tracheostomy *n* (%)	Overall mortality *n* (%)
Holscher et al.^25^	2014	91	–	67 (73.6)	–	2 (2.2)
Ogilvie et al.^27^	2014	231	111 (48.0)	154 (66.7)	5 (2.2)	37 (16.0)
Pérez-Ruiz et al.^16^	2012	249	150 (60.2)	150 (60.2)	8 (3.2)	31 (12.4)
Trey et al.^8^	2012	119	83 (69.7)	70 (58.8)	1 (0.8)	25 (21.0)
Wood et al.^9^	2012	1613	739 (45.8)	932 (57.8)	–	90 (5.6)
Atmaca et al.^6^	2011	54	26 (48.1)	32 (59.3)	–	15 (27.8)
Itamoto et al.^5^	2010	58	–	33 (56.9)	2 (3.4)	2 (3.4)
Al-Samri et al.^14^	2010	72	–	43 (59.7)	1 (1.4)	11 (15.3)
Adoga e Ma’na^10^	2010	46	–	29 (63.0)	–	8 (17.4)
Berry et al.^34^	2009	917	524 (57.1)	541 (59.0)	11 (1.2)	71 (7.7)
Gutiérrez-Gutiérrez et al.^3^	2009	50	2 (4.0)	33 (66.0)	–	11 (22.0)
Ramku et al.^35^	2009	30		20 (66.7)	–	6 (20.0)
Ozmen S et al.^1^	2009	282	23 (8.2)	181 (64.2)	3 (1.1)	56 (19.8)
Karapinar B et al.^24^	2008	31	–	12 (38.7)	1 (3.2)	10 (32.3)
Graf et al.^36^	2008	70	–	43 (61.4)	–	9 (12.8)
Corbett et al.^17^	2007	112	58 (51.8)	–	2 (1.8)	22 (19.6)
Parrilla et al.^15^	2007	38	19 (50.0)	20 (52.6)	–	15 (39.5)
Mahadevan et al.^2^	2007	122	81 (66.4)	67 (54.9)	2 (1.6)	17 (14.0)
Rozsasi et al.^11^	2005	24	–	19 (79.2)	1 (4.2)	6 (25.0)
Butnaru^37^	2005	46	16 (34.8)	24 (52.2)	1 (2.2)	6 (13.0)
Ang et al.^7^	2005	48	30 (62.5)	29 (60.4)	–	8 (16.7)
Solares et al.^38^	2004	94	60 (63.8)	47 (50.0)	–	8 (8.5)
Tantinikorn et al.^18^	2003	181	99 (54.7)	108 (59.7)	1 (0.55)	24 (13.3)
Midwinter et al.^39^	2002	143	93 (65.0)		4 (2.8)	
Ilçe et al.^26^	2002	17	7 (41.2)	13 (76.5)	1 (5.9)	10 (58.8)
Rocha et al.^33^	2000	46	–	35 (76.1)	–	6 (13.0)
Wetmore et al.^23^	1999	373	292 (78.3)	253 (67.8)	2 (0.54)	94 (25.2)
Dubey et al.^40^	1999	40	13 (32.5)		1 (2.5)	10 (25.0)
Koltai^41^	1998	68	43 (63.2)	36 (52.9)	–	7 (10.3)
Donelly et al.^12^	1996	29	14 (48.3)	15 (51.7)	–	6 (20.7)
Shinkwin e Gibbin^28^	1996	56	40 (71.4)	29 (51.8)	1 (1.8)	8 (14.3)
Ward^29^	1995	103	64 (62.1)	65 (63.1)	3 (2.9)	37 (35.9)
Freezer et al.^13^	1990	142	70 (49.3)	82 (57.7)	2 (1.4)	19 (13.4)
Simma et al.^42^	1994	108	64 (59.3)		–	8 (7.4)
Zeitouni et al.^43^	1993	44	44 (100)		2 (4.5)	
Puhakka et al.^44^	1992	33		13 (39.4)	–	5 (15.1)
Line^45^	1986	153			5 (3.3)	34 (22.2)

*Note*: Dashes represent “0”. Blank spaces represent a lack of information in the article.

When the decades were considered individually, the frequency of children younger than one year of age ranged from 41.3% (1985–1994) to 63.0% (1995–2004), with a statistically significant increase in the last decade relative to the other decades (*p* < 0.001).

There was a progressive increase in the frequency of male children undergoing tracheostomies, with a statistically significant increase between the decades evaluated (Table 3).

**Table 3:** Epidemiological data on children who have received a tracheostomy between 1985 and 1994, between 1995 and 2004, and between 2005 and 2014.

	Decade *n* (%)			*p*-Value^a^		
	1985–1994	1995–2004	2005–2014	1985–1994 vs. 1995–2004	1985–1994 vs. 2005–2014	1995–2004 vs. 2005–2014
	*n* = 480	*n* = 1150	*n* = 4303			
<1 year of age	198 (41.3)	725 (63.0)	1862 (43.3)	<0.001^b^	0.396	<0.001^b^
Male	95 (19.8)	601 (52.3)	2499 (58.1)	<0.001^b^	<0.001^b^	<0.001^b^

a Chi-square test.

b *p* < 0.05.

### Complications

Early post-operative complications and late post-operative complications were evaluated as a set. Excluding the studies that considered only premature and/or extremely underweight newborns, the most common complications during the entire period in descending order of frequency were granuloma, infection, obstruction of the cannula, and in fourth place, accidental decannulation and a post-decannulation tracheocutaneous fistula, as shown in Table 4.

**Table 4:** Complications in children who received a tracheostomy between 1985 and 1994, between 1995 and 2004, and between 2005 and 2014.

Complication	Decade *n* (%)				*p*-Value^a^		
	1985–1994	1995–2004	2005–2014	Total	1985–1994 vs. 1995–2004	1985–1994 vs. 2005–2014	1995–2004 vs. 2005–2014
	*n* = 480	*n* = 1150	*n* = 4303	*n* = 5933			
Obstruction of the cannula	20 (4.2)	87 (7.6)	132 (3.1)	239 (4.0)	0.012^b^	0.193	<0.001^b^
Accidental decannulation	15 (3.1)	60 (5.2)	112 (2.6)	187 (3.1)	0.066	0.004^b^	<0.001^b^
Bleeding	3 (0.6)	5 (0.4)	29 (0.7)	37 (0.6)	0.616	0.901	0.36
Subcutaneous mphysema	–	7 (0.6)	8 (0.2)	15 (0.2)	–	–	0.015^b^
Pneumothorax	27 (5.6)	15 (1.3)	27 (0.6)	69 (1.2)	<0.001^b^	<0.001^b^	0.02^b^
Pneumomediastinum	15 (3.1)	11 (1.0)	13 (0.3)	39 (0.7)	0.001^b^	<0.001^b^	0.003^b^
Granuloma	14 (2.9)	212 (18.4)	206 (4.8)	432 (7.3)	<0.001^b^	0.063	<0.001^b^
Tracheomalacia	1 (0.2)	7 (0.6)	10 (0.2)	18 (0.3)	0.292	0.917	0.042^b^
Infection	1 (0.2)	122 (10.6)	194 (4.5)	317 (5.3)	<0.001^b^	0.453	<0.001^b^
Stromal collapse	–	–	14 (0.3)	14 (0.2)	–	–	–
Post-decannulation tracheocutaneous fistula	13 (2.7)	121 (10.5)	53 (1.2)	187 (3.1)	<0.001^b^	0.009^b^	<0.001^b^
Stenosis	12 (2.5)	7 (0.6)	60 (1.4)	79 (1.3)	0.001^b^	0.059	0.032^b^

*Note*: Dashes represent “0,” “not reported” or “statistical analysis not possible”.

a Chi-square test.

b *p* < 0.05.

All of these most common complications (granuloma, infection, obstruction of the cannula, accidental decannulation, and post-decannulation tracheocutaneous fistula) occurred most frequently in the studies published between 1995 and 2004. Granuloma, infection, tracheocutaneous fistula, and obstruction of the cannula presented a significant difference in this decade relative to the others (*p* < 0.05). Decannulation, meanwhile, differed significantly only between the 1995–2004 period and the 2005–2014 period (*p* < 0.001); there was a significant reduction in the third decade relative to the first and second decades (*p* = 0.004 and *p* < 0.001).

Tracheomalacia and subcutaneous emphysema were also more frequent between 1995 and 2004; as shown in Table 4, both frequencies were statistically significant relative to the third decade (*p* = 0.015 and *p* = 0.042, respectively).

Meanwhile, pneumothorax, pneumomediastinum, and stenosis were the most frequent complications from 1985 to 1994; the frequency of pneumothorax and pneumomediastinum differed significantly in this decade relative to the other 2 (*p* < 0.001 and *p* = 0.001). Stenosis frequency differed significantly between the 1985–1994 period and the 1995–2004 period (*p* = 0.001).

Between 2005 and 2014, bleeding and stromal collapse were the most frequent complications; however, the frequency of bleeding did not differ significantly among the periods considered (*p* = 0.616 between the first decade and second decade; *p* = 0.901 between the first decade and the third decade, and *p* = 0.360 between the second decade and the third decade). Stromal collapse was a rare complication, occurring in 0.3% of patients and present only in the 2005–2014 period.

### Mortality

Among the studies considered from these three decades (with the exception of those that reported only on premature and/or extremely underweight newborns), mortality associated with tracheostomy ranged from 0 to 5.9%,^26^ while overall mortality ranged from 2.2% to 59% (Table 2).^19^, ^20^, ^21^, ^22^, ^25^, ^26^ Mortality associated with the procedure decreased from 2.1% between 1985 and 1994 to 0.9% between 2005 and 2014 (*p* = 0.012). Meanwhile, overall mortality was higher between 1995 and 2004 than in the other decades (*p* < 0.05). The data on mortality associated with tracheostomy and on overall mortality are outlined in Table 5.

**Table 5:** Mortality in children who received a tracheostomy between 1985 and 1994, between 1995 and 2004, and between 2005 and 2014.

Mortality	Decade *n* (%)			*p*-Value^a^		
	1985–1994	1995–2004	2005–2014	1985–1994 vs. 1995–2004	1985–1994 vs. 2005–2014	1995–2004 vs. 2005–2014
	*n* = 480	*n* = 1150	*n* = 4303			
Mortality associated with tracheostomy	10 (2.1)	13 (1.1)	38 (0.9)	0.137	0.012^b^	0.439
Overall mortality	66 (13.8)	210 (18.3)	458 (10.6)	0.027^b^	0.039^b^	<0.001^b^

a Chi-square test.

b *p* < 0.05.

### Premature and/or extremely underweight newborns

The details from the studies that considered only premature and/or underweight children are reported in Table 6. Overall mortality in this group ranged from 8.2% to 44.4%, while in the rest of the studies, overall mortality of children who had received a tracheostomy ranged from 2.2% to 59%. Mortality associated to tracheostomy was not reported in this group.

**Table 6:** Mortality rates in premature and/or extremely underweight children who received a tracheostomy between 1985 and 2014.

	Year of publication	*n*	Mortality associated with tracheostomy *n*(%)	Overall mortality *n*(%)
DeMauro et al.^19^	2014	304	–	25 (8.2)
Viswanathan et al.^20^	2013	9	–	4 (44.4)
Sidman et al.^22^	2006	78	–	12 (15.4)
Pereira et al.^21^	2004	55	–	9 (16.4)

*Note*: Dashes represent “0” or “not reported”.

## Discussion

The objective of this review was to evaluate the occurrence of complications and of mortality associated with tracheostomy over the last three decades. Many children younger than one year of age who had undergone a tracheostomy were found to be included in these studies; rates of this age group varied from 41.3% to 63.0%. This finding reflects the trend of progressively younger children receiving this procedure, as was observed in many studies.^6^, ^10^, ^15^, ^23^, ^27^, ^39^, ^46^, ^47^ This age distribution may be attributed to advances in intensive care techniques, changes in the epidemiology of infectious diseases, and the increase in survival rates of premature newborns and of newborns born with birth defects.^2^, ^10^, ^11^, ^12^, ^13^, ^14^, ^15^, ^16^ Tracheostomies were predominantly performed in male children, perhaps because males are more susceptible to both congenital and acquired defects.^10^, ^44^

Tracheostomy in children is associated with higher rates of complications than tracheostomy in adults.^1^, ^2^, ^4^, ^5^, ^7^, ^10^, ^15^, ^17^, ^18^, ^24^, ^25^, ^28^, ^29^ The highest rates of pneumothorax and pneumomediastinum occurred between 1985 and 1994, with a significant reduction in the following two decades. These complications occur due to damage to the pleura during the procedure, since pleural apices are found at a higher position in children than in adults. They may extend into the inferior portion of the neck, which makes complications more likely.^30^ The drop in these complications in recent years may be explained by the decrease in tracheostomies performed in patients with any kind of airway infection, as well as by the greater frequency of the procedure being performed in the operating room and under general anesthesia.^31^

In the 1995–2005 period, the main complication observed was granuloma followed by infection, tracheocutaneous fistula, and obstruction of the cannula. This data is inconsistent with the studies from this decade that report the most common complications to be obstruction and decannulation.^23^, ^28^, ^30^ However, these complications are considered more important among those that precede or are most highly associated with death, and the present study did not analyze the frequency of early and late post-operative complications separately because there was no distinction in the literature to allow for this evaluation. It was also for this same reason that the causes of morality directly associated with tracheostomy were not evaluated. The frequency of granuloma was also considered. Some authors argue that granuloma should not be consider as complication of tracheostomy, but instead an expected result.^27^ However, among those who considered it to be a complication, granuloma was one of the most common.^1^, ^2^, ^4^, ^8^, ^14^, ^26^

Between 2005 and 2014, granulomas were found to be the most common complication, followed by infection, obstruction of the cannula, and accidental decannulation. A single study that evaluated a population of 72 patients between 1990 and 2007 represented 62 of the 206 of granuloma 30.1%) and 87 of the 194 of infection (44.8%).^14^ This study defined infection as the increase in secretions by the tracheostomy, increased neutrophil counts in the secretion, and/or positive cultures. The authors defended the use of medication as treatment in cases in which it was impossible to distinguish between colonization and infection. This may have influenced the order of the most important complications in this decade. In the review of the literature, infection increased significantly in the second decade relative to the first and third decades (*p* < 0.001). It was the second most common complication in the 1995–2004 period, during which time it represented 10.6% of cases. It is important to note that, in this review, the terms “operative wound infection”, “stroma infection”, “abscess”, “cellulitis”, “tracheitis”, and “pneumonia” were considered as a set; in some articles, they were considered individually. However, some authors emphasize that there needs to be a distinction between infection and colonization at the tracheostomy site.^30^ Children who use a tracheostomy for an extended period of time are more frequently colonized by *Pseudomonas aeruginosa* and/or *Staphylococcus aureus*, which suggests that the colonization does not require treatment unless there are signs of infection. Unfortunately, this distinction was not possible in this paper because of insufficient or inadequate data.

Obstruction of the cannula was most frequent in the articles published between 1995 and 2004; it increased in this period relative to the other two decades evaluated. However, there was no significant decrease relative to the first or third decades (*p* = 0.193). During this 30-year period, it was therefore among the four most common complications of tracheostomy when both early and late post-operative complications were considered. The frequency of accidental decannulation decreased significantly in the third decade relative to the first and second decades (*p* = 0.004 and *p* < 0.001), though it still remained among the five most common complications during the period studied. The cannula obstruction is a common problem and its high frequency in younger children is likely associated with the narrow inner radius of small tracheostomy cannulas, as well as with the common condition of bronchopulmonary dysplasia, which is itself associated with viscous bronchial secretions in premature newborns.^15^, ^32^ It has been believed that both obstruction and decannulation are complications that can be avoided with adequate tracheostomy tube care.^30^ The continual administration of humidified air should be performed until the first cannula change in order to prevent its obstruction. In addition, the entire team involved in the tracheostomy patient's care should be trained to know how to handle obstruction and accidental decannulation, as well as emergency cannula changes and even CPR, in order to avoid catastrophic consequences when these events occur.

Stenosis was a complication that did not differ significantly when the 1985–1994 period was compared to the 2005–2014 period (*p* = 0.059). Though the operative technique has changed over the years, some authors report that there is a debate in the literature regarding the formation of tracheal stenosis that depends on the technique used; however, no consensus has been reached.^29^, ^30^, ^31^ Most studies do not report this association, despite the fact that animal studies have confirmed it.^31^ This complication should be considered even in extremely underweight newborns, and there has been reported that the larger the tracheostomy tube, the more common stenosis is.^20^

The tracheocutaneous fistulas were significantly less frequent in the third decade relative to the first two decades under study; between 2005 and 2014, they were present in 1.2% of cases. However, they were most frequent during the second decade under study. An hypothesis is that prolonged use of a tracheostomy cannula may cause the stroma to decrease to a non-functional size, though it does not close completely; this change leads to the formation of a tracheocutaneous fistula and some authors consider that tracheocutaneous fistulas may be more common depending on the technique used for the tracheostomy.^15^, ^18^, ^30^ One example is the starplasty technique, which attaches the stroma to the skin using sutures in order to reduces indices of pneumothorax and accidental decannulation, but it often requires later intervention to correct the fistula, which is expected.^38^, ^41^, ^46^

Only four studies specifically focused on premature and/or extremely underweight newborns who received tracheostomy were selected from the literature of the last thirty years. Thus, there were limits to the evaluation of this group in terms of the complications and mortality these patients experience. However, some studies have noted that this group is more susceptible to higher rates of both complications and mortality due to the conditions presented at birth, which also contribute to a higher rate of mortality associated with the surgical procedure.^29^

Various studies have demonstrated that many complications associated with tracheostomy in children may be avoided by the use of specific surgical techniques, of adequate tubes, and of careful maneuvers to maintain the tracheostomy and to remove cannulas.^11^, ^33^, ^41^, ^46^

Mortality associated with tracheostomy is higher in the pediatric age group than among adults^1^, ^2^, ^4^, ^5^, ^7^, ^10^, ^15^, ^17^, ^18^, ^24^, ^25^, ^28^; however, most pediatric deaths are associated not with the procedure itself, but with underlying diseases. Morbidity and mortality rates depend significantly on how well informed and trained the medical team, the patients’ parents, and the patients’ caregivers are.^2^, ^4^, ^7^, ^17^ Furthermore, the association between lower indices of complications and death with the performance of the procedure in hospitals of reference by surgeons trained in the management of airway obstruction in children was found.^4^ This analysis of the literature revealed that, among the studies that evaluated all pediatric age groups, there was a significant decrease in morbidity and mortality between the 1985–1994 period and the 2005–2014 period (*p* = 0.012); the frequency fell from 2.1% to 0.9%. There was a significant difference in mortality rates between each of the decades, with the highest number of deaths between 1995 and 2004. When only the articles that exclusively analyzed premature and/or extremely underweight newborns were considered, a significant increase in overall mortality was found in the data from 2006 relative to the data from 2013 (*p* = 0.033), as was a significant decrease in overall mortality in the data from 2013 relative to 2014.^19^, ^20^, ^21^, ^22^ None of these studies reported mortality to be directly associated with tracheostomy, though the literature reports higher morbidity and mortality rates in this patient group, as well as among children under one year of age.^4^, ^7^, ^15^, ^17^, ^28^, ^29^, ^33^

## Conclusion

In this review of the literature, we found that complications and mortality rates of tracheostomy have undergone certain changes over the last three decades. The complications that were previously the most frequent were pneumothoraces and pneumomediastinum; today, these complications have become less common. Meanwhile granuloma, infection, and obstruction of the cannula are the most common complications experienced by patients who have received a tracheostomy. Accidental decannulation decreased considerably in the in the last decade under study. During this last decade, we also observed a decrease in the mortality rate associated with the procedure.

## Conflicts of interest

The authors declare no conflicts of interest.
